# PGC-1α regulates airway epithelial barrier dysfunction induced by house dust mite

**DOI:** 10.1186/s12931-021-01663-6

**Published:** 2021-02-19

**Authors:** Tsutomu Saito, Tomohiro Ichikawa, Tadahisa Numakura, Mitsuhiro Yamada, Akira Koarai, Naoya Fujino, Koji Murakami, Shun Yamanaka, Yusaku Sasaki, Yorihiko Kyogoku, Koji Itakura, Hirohito Sano, Katsuya Takita, Rie Tanaka, Tsutomu Tamada, Masakazu Ichinose, Hisatoshi Sugiura

**Affiliations:** 1 Department of Respiratory Medicine, Tohoku University Graduate School of Medicine, 1-1 Seiryo-machi, Aoba-ku, Sendai, 980-8574 Japan; 2 Department of Respiratory Medicine, Sendai City Hospital, Sendai, Japan; 3 Department of Respiratory Medicine, Osaki Citizen Hospital, Osaki, Miyagi Japan

**Keywords:** Asthma, Airway barrier dysfunction, PGC-1α, House dust mite

## Abstract

**Background:**

The airway epithelial barrier function is disrupted in the airways of asthmatic patients. Abnormal mitochondrial biogenesis is reportedly involved in the pathogenesis of asthma. However, the role of mitochondrial biogenesis in the airway barrier dysfunction has not been elucidated yet. This study aimed to clarify whether the peroxisome proliferator-activated receptor γ coactivator-1alpha (PGC-1α), a central regulator of mitochondrial biogenesis, is involved in the disruption of the airway barrier function induced by aeroallergens.

**Methods:**

BEAS-2B cells were exposed to house dust mite (HDM) and the expressions of PGC-1α and E-cadherin, a junctional protein, were examined by immunoblotting. The effect of SRT1720, a PGC-1α activator, was investigated by immunoblotting, immunocytochemistry, and measuring the transepithelial electrical resistance (TEER) on the HDM-induced reduction in mitochondrial biogenesis markers and junctional proteins in airway bronchial epithelial cells. Furthermore,the effects of protease activated receptor 2 (PAR2) inhibitor, GB83, Toll-like receptor 4 (TLR4) inhibitor, lipopolysaccharide from Rhodobacter sphaeroides (LPS-RS), protease inhibitors including E64 and 4-(2-Aminoethyl) benzenesulfonyl fluoride hydrochloride (AEBSF) on the HDM-induced barrier dysfunction were investigated.

**Results:**

The amounts of PGC-1α and E-cadherin in the HDM-treated cells were significantly decreased compared to the vehicle-treated cells. SRT1720 restored the expressions of PGC-1α and E-cadherin reduced by HDM in BEAS-2B cells. Treatment with SRT1720 also significantly ameliorated the HDM-induced reduction in TEER. In addition, GB83, LPS-RS, E64 and AEBSF prevented the HDM-induced reduction in the expression of PGC1α and E-cadherin.

**Conclusions:**

The current study demonstrated that HDM disrupted the airway barrier function through the PAR2/TLR4/PGC-1α-dependent pathway. The modulation of this pathway could be a new approach for the treatment of asthma.

## Background

Asthma is a chronic airway inflammatory disease that is characterized by variable symptoms of wheeze, shortness of breath, chest tightness and/or cough, and by variable expiratory airflow limitation [1]. Although it is considered a heterogenous disease with multiple molecular mechanisms, the airway inflammation of asthma is generally associated with allergic mechanisms related to type 2 airway inflammation. Aeroallergens such as house dust mite (HDM), animal dander, fungal spores, plant and tree pollen play a pivotal role in the pathophysiology of allergic asthma because they trigger immune responses with the activation of allergen-specific T helper type 2 (Th2) cells [2–4]. The bronchial epithelium forms a physical barrier against the external environment through the formation of cell–cell junctions consisting of tight junctions, adherence junctions and desmosomes, referred to as the epithelial junctional complex [5, 6]. Tight junctions and adherence junctions are located at the apical end of the lateral membrane and are comprised of numerous membrane proteins including occludin, zonula occludens (ZO)-1 and E-cadherin [5, 6]. A reduction in the expression of E-cadherin and ZO-1 is reportedly observed in the airways of asthmatic patients [7, 8]. Allergens with proteolytic activity such as HDM can directly cleave epithelial tight junctions and disrupt barrier structures [9, 10]. The major HDM, *Dermatophagoides pteronyssinus* allergen Der p 1 is known to cleave tight junctions directly and indirectly through protease-activated receptor-2 activation [11]. Disruption of the epithelial barrier increases the susceptibility to external stimuli leading to airway hyperresponsiveness. Furthermore, a damaged epithelial barrier increases the accessibility of allergens into the submucosa activating the subsequent immune responses. Thus, regulation of the bronchial epithelial function has been attracting attention as an important immunological checkpoint in asthma. However, the precise mechanisms by which epithelial junctions are disrupted are not fully understood.

In airway epithelial cells and BEAS-2B cells, interleukin (IL)-4 reportedly promotes intracellular asymmetric dimethylarginine (ADMA) accumulation, which causes a reduction in mitochondrial biogenesis [12]. Though the result of the mitochondrial biogenesis reduction is unknown, since most important role of airway epithelial cells is the airway barrier function, it is probable that the reduction affects airway the barrier disfunction.

Mitochondria play a key role in energy homeostasis and the metabolism of reactive oxygen species (ROS) [13]. Appropriate elimination of damaged mitochondria through mitochondrial autophagy (mitophagy) and the renewal of mitochondria by mitochondrial biogenesis are essential for mitochondrial homeostasis [14]. Mitochondrial biogenesis is regulated mainly at the transcriptional level and requires the coordinated expression of both nuclear-encoded and mitochondrial-encoded proteins, including peroxisome proliferator-activated receptor γ coactivator-1α (PGC-1α), mitochondrial transcriptional factor A (TFAM), adenosine 5′‑monophosphate‑activated protein kinase (AMPK), and nuclear respiratory factors (NRF)-1 and -2 [14]. Among these molecules, PGC-1α is the key regulator of mitochondrial biogenesis [15].

Sirtuin 1 (SIRT1) is a powerful deacetylase that has been shown to activate PGC-1α to drive mitochondrial biogenesis [16], and SRT1720, the activator of SIRT1, is an effective SIRT1 agonist that enhances PGC-1α activation [17–19].　In previous reports, SRT1720 alleviated lung injury and improved the lung function in rat with emphysema caused by cigarette smoke through protecting against the apoptosis of type II alveolar epithelial cells [20]. SRT1720 inhibited the differentiation of TGF-β1-induced myofibroblasts [21]. SRT1720 repressed the LPS-induced release of cytokines such as IL-8, IL-6 and tumor necrosis factor (TNF)-α from cultured peripheral blood mononuclear cells [22]. In a report about asthma, SRT1720 also suppressed inflammatory cell infiltration and cytokine production including TNF-α and IL-6 in the lungs of an ovalbumin (OVA)-induced mouse model [23]. It is probable that the activation of mitochondrial biogenesis by SRT1720 in airway epithelial cells contributes to amelioration of the asthma pathophysiology.

Therefore, the current study aimed to clarify the contribution of regulators of mitochondrial biogenesis to airway barrier dysfunction. We assessed the effect of house dust mite (HDM), a common aeroallergen related to asthma, on the expression of mitochondrial biogenesis markers and junctional proteins in airway epithelial cells. Furthermore, we investigated how an activator of PGC-1α modulates the constitution of junctional proteins and the airway barrier function to explore novel therapeutic targets for bronchial asthma.

## Materials and methods

### Materials

The following reagents were used in this study: purified HDM extract from *Dermatophagoides pteronyssinus* was purchased from LSL (Tokyo, Japan); SRT1720 was from Selleck Chemicals (Houston, TX); GB83 was from Axon Medchem (Groningen, Netherlands); Lipopolysaccharide from *Rhodobacter sphaeroides* (LPS-RS) was from Invivogen (San Diego, CA); Dexamethasone, E64, 4-(2-Aminoethyl) benzenesulfonyl fluoride hydrochloride (AEBSF), and mouse monoclonal anti-β-actin antibody were from Sigma (St Louis, MO). Protein block, a blocking reagent, was from Dako (Kyoto, Japan); Rabbit polyclonal anti-PGC-1α antibody, rabbit monoclonal anti-TFAM antibody, rabbit monoclonal anti-PINK1 antibody, rabbit monoclonal anti-E-cadherin antibody, rabbit polyclonal anti-ZO-1 antibody, FITC-conjugated goat anti-rabbit secondary antibody, and Alexa Fluor 647-conjugated goat anti-rabbit secondary antibody were from Abcam (Cambridge, MA); horseradish peroxidase–conjugated secondary antibodies and mouse monoclonal anti-inducible nitric oxide synthases (iNOS) antibody were from Santa Cruz Biotechnology (Dallas, TX); MitoTracker Red probe and Hoechst 33,342 were from Invitrogen Life Technologies (Eugene, OR); Keratinocyte-SFM (serum-free medium) and Human Keratinocyte Growth Supplements were from Gibco (Grand Island, NY). Bronchial epithelial growth medium (BEGM) bullet kit including bronchial epithelial basal medium (BEBM) and subculture reagents were purchased from Lonza Japan Bioscience (Tokyo, Japan).

### Cell culture

BEAS-2B cells, a human bronchial epithelial cell line, were obtained from ﻿American Type Culture Collection (Manassas, VA). The BEAS-2B cells were cultured in Keratinocyte-SFM medium supplemented with Keratinocytes Supplements at 37 ℃ in a humidified atmosphere of 5% CO_2_ and passaged in 75 cm^2^ flasks. Normal primary human bronchial epithelial cells (pHBEC) were obtained from Lonza (Wokingham, UK) and cultured in BEGM. To prepare BEGM, BEBM was supplemented with human recombinant epidermal growth factor (0.5 ng/ml), insulin (5 μg/ml), transferrin (10 μg/ml), hydrocortisone (0.5 μg/ml), triiodothyronine (6.5 μg/ml), epinephrine (0.5 μg/ml), retinoic acid (50 nM), gentamycin and amphotericin-B (50 μg/ml) and bovine pituitary extract (35 mg/ml).

### Animals

8-week-female C57BL/6 J mice were purchased from SEIMI (Sendai, Japan). All mice were housed in a specific pathogen-free facility and maintained under constant temperature (24 °C), humidity (40%), and light cycle (8:00 A.M. to 8:00 P.M.), with food and water provided ad libitum. The mice were exposed to HDM (30 μg/body, 50 μl) intranasally every other day as described previously [24]. The control mice received PBS. The mice were euthanized on day 15, and the trachea was cannulated after the pulmonary circulation was perfused and free of blood. The lung was inflated with 10% paraformaldehyde for 10 min at 25 cm H_2_O and subsequently removed and fixed in 10% paraformaldehyde for 24 h at room temperature. Samples were then dehydrated in ethanol and xylene, embedded in paraffin, cut in 5-μm sections. All experiments were approved by the Tohoku University Animal Experiment Ethics Committee and performed in accordance with the Regulations for Animal Experiments and Related Activities at Tohoku University.

### Western blotting

The expression of markers of mitochondrial biogenesis (PGC-1α and TFAM) and junctional proteins (E-cadherin and ZO-1) in BEAS-2B cells was analyzed by western blotting. Cells (2 × 10^5^ cells/ml) were seeded in 6-well culture plates and grown to 80–90% confluence. Then the cells were maintained in Keratinocytes Supplements free medium for 24 h and stimulated with or without HDM (100 μg/ml). 24 h after HDM stimulation, the cells were homogenized in cell lysis buffer (0.05% TritonX, 3 mM Tris–HCl, pH7.4 0.4 mM EGTA, 10 mM MgCl2, 1 μM phenylmethylsulfonyl fluoride, 100 μg/ml aprotinin, 1 μg/ml leupeptin) on ice and harvested for western blotting. SRT1720 (1 μM), a PGC-1α activator, was added to the culture medium at 6 h before HDM stimulation. Protease activated receptor (PAR) 2 inhibitor GB83, Toll-like receptor (TLR) 4 inhibitor LPS-RS, cysteine peptidase inhibitor E64, and serine peptidase inhibitor AEBSF were added at 30 min before HDM stimulation. Synthetic glucocorticoid dexamethasone was added at 24 h before HDM stimulation. The whole cell lysates were homogenized for several seconds using an ultrasonic homogenizer (BANDELIN, Berlin, Germany). The samples were solubilized in SDS-PAGE sample buffer (Bio-Rad, Hercules, CA). Equal amounts of protein were loaded and separated by electrophoresis on 12% SDS polyacrylamide gels. After electrophoresis, the separated proteins were transferred to polyvinylidene difluoride membranes (Millipore, Darmstadt, Germany). The membranes were blocked with a blocking reagent. Rabbit polyclonal anti-PGC-1α antibody (1:5000), rabbit monoclonal anti-TFAM antibody (1:5000), rabbit monoclonal anti-PINK1 antibody (1:1000), rabbit monoclonal anti-E-cadherin antibody (1:10,000), rabbit polyclonal anti-ZO-1 antibody (1:2000), mouse monoclonal anti-iNOS antibody (1:200) and mouse monoclonal anti-β-actin antibody (1:5000) were used as primary antibodies to detect the target proteins. Horseradish peroxidase–conjugated secondary antibodies (1:5000) were detected using ECL-prime Western Blotting Reagent (Amersham Biosciences, Buckinghamshire, UK) and visualized with a chemiluminescence imaging system (LAS-4000 mini, FUJIFILM, Tokyo, Japan). Each band intensity was quantified using ImageJ (National Institutes of Health, Bethesda, MD, USA).

### Evaluation of mitochondrial mass and immunochemical localization of E-cadherin and ZO-1 in BEAS-2B cells

BEAS-2B cells were seeded in 8-well chamber slides at a density of 1 × 10^5^ cells/ml and cultured for 24 h, and then the media were replaced with the keratinocyte supplement-free medium for 24 h. After incubation with 1 µM SRT1720 for 6 h, the cells were stimulated with HDM (100 μg/ml) for 24 h. The mitochondrial mass was evaluated using MitoTracker Red probe. MitoTracker Red was added to the existing medium to a final concentration of 200 nM and cells were incubated for 30 min at 37 ℃, 5% CO_2_. After staining, the cells were washed once with PBS. Then, the slides were fixed with freshly prepared 4% paraformaldehyde in PBS for 30 min at room temperature. The slides were permeabilized with 0.1% Triton X-100 in PBS for 10 min at room temperature. The slides were blocked with a blocking solution for 30 min at room temperature. The slides were then incubated overnight with rabbit monoclonal anti-E-cadherin antibody (1:500), rabbit polyclonal anti-ZO-1 antibody (1:200) at 4℃. The slides were incubated with the appropriate FITC-conjugated secondary antibody (1:3000) for 1 h at room temperature. The nuclei of the cells were stained with Hoechst 33,342 (1:200). Fluorescent images were obtained using a confocal laser scanning microscope system (Nikon ECLIPSE Ti-E, C2si; Nikon, Tokyo, Japan). The mean fluorescence intensity of E-cadherin, ZO-1, MitoTracker were quantified by using ImageJ.

### Immunochemical localization of PGC-1α, TFAM and E-Cadherin in lung tissues from mice

Tissue samples were fixed in 10% formalin and embedded in paraffin. 5 μm thick serial tissue sections were obtained and mounted in Superfrost/Plus glass slides (Fischer Scientific). Deparaffinization was performed by washing three times for 5 min in xylene, then washing in 100%, 95%, 80%, 70% ethanol three times for 5 min and, finally, rinsing with distilled water. Slides were washed in PBS for 5 min and processed for antigen retrieval using citrate buffer. The sections were permeabilized with Triton-X 100 for 15 min. Sections were then washed in PBS, blocked using blocking reagent (Dako) for 60 min at room temperature. Sections were incubated overnight at 4 °C with rabbit polyclonal anti-PGC-1α antibody (1:1000), rabbit monoclonal anti-TFAM antibody (1:10,000), rabbit monoclonal anti-E-Cadherin antibody (1:10,000) or nonspecific polyclonal rabbit IgG as a negative control at 4 °C overnight. After being washed, the samples were incubated with Alexa Fluor 647-conjugated goat anti-rabbit secondary antibody (1:3000) for 60 min at room temperature. Omission of the primary antibody was used as negative control. After washing, the nuclei of the cells were stained with Hoechst 33,342 (1:200). Fluorescent images were obtained by microscopy (BX53-33-SDO; Olympus, Tokyo, Japan) and photographed with a digital camera (DP71-SET; Olympus). The mean fluorescence intensities of PGC-1α, TFAM, and E-cadherin were quantified by using ImageJ.

### Quantification of interleukin (IL)-33 in supernatant of BEAS-2B cells

The amounts of IL-33 in the supernatants of BEAS-2B cells were determined using DuoSet Kit (R&D Systems, Minneapolis, MN, USA) according to the manufacturer’s instructions.

### Transepithelial electrical resistance measurements (TEER)

Primary human bronchial epithelial cells were seeded onto collagen type I coated 12-well transwell inserts (transparent, 0.4 µm; Greiner) at 1 × 10^5^ cells/cm^2^ with 500 µl apical and 1500 µl basolateral volumes, maintained in BEGM, and incubated until the formation of cell monolayers. Then, SRT1720 (1 µM) was added to the apical medium. After 24 h incubation with SRT1720, the cells were stimulated with HDM (100 ng/ml) or DMSO (vehicle) for 24 h. Then, TEER was monitored using a Millicell ERS-2 voltohmmeter (Millipore, Billerica, MA).

### Statistical analysis

Results are presented as mean ± SD. Comparisons among groups were performed by One-way analysis of variance followed by the Tukey's multiple comparison test or unpaired t-test. An unpaired two-tailed Student t test was used for single comparisons. p < 0.05 was considered statistically significant.

## Results

### Expression of mitochondrial biogenesis markers and junctional proteins in HDM-exposed BEAS-2B cells

**Fig. 1:**
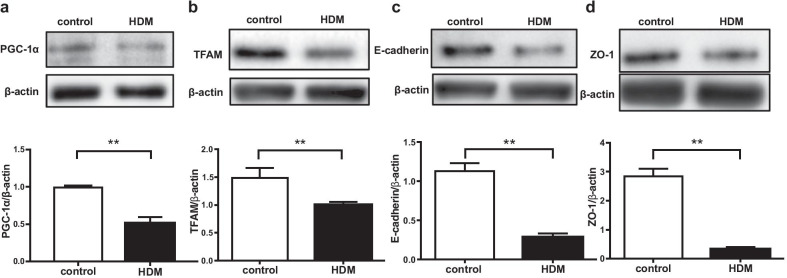
Expression of mitochondrial biogenesis markers and junction proteins in bronchial epithelial cells. **a**–**d** BEAS-2B cells were treated with HDM (100 μg/ml) or vehicle for 24 h and the cells were harvested. Expressions of PGC-1α, TFAM, E-cadherin and ZO-1 were analyzed by western blotting. Data are expressed as means ± SD (n = 3). *p < 0.05, **p < 0.01

Because HDM is a common aeroallergen in asthmatic patients, we investigated the effect of HDM on the expression of mitochondrial biogenesis makers and junctional proteins in BEAS-2B cells. The cells were treated with HDM at various concentrations and time points. HDM significantly reduced the expression of TFAM in a concentration-dependent (Additional file 1: Figure S1a) and time-dependent manner (Additional file 1: Figure S1b). In the HDM-treated cells, the expression of markers of mitochondrial biogenesis was significantly reduced compared to the control cells (PGC-1α, *p* < 0.01; TFAM, *p* < 0.01) (Fig. 1a, b). The expression of junctional proteins was also significantly reduced compared to the control cells (E-cadherin, *p* < 0.01; ZO-1, *p* < 0.01) (Fig. 1c, d). We also investigated the effect of HDM on PINK1 expression, a marker for mitophagy, in the cells. HDM administration significantly increased the protein level of PINK1 compared to controls in BEAS-2B cells (Additional file 1: Figure S2a). As oxidative and nitrative stress reportedly play an important role in HDM-induced asthma [25], we determined iNOS expression in the HDM-treated cells. HDM significantly increased iNOS expression in the cells (Additional file 1: Figure S2b).

### Expressions of mitochondrial biogenesis markers and junctional proteins in the lungs of HDM-treated mice

**Fig. 2:**
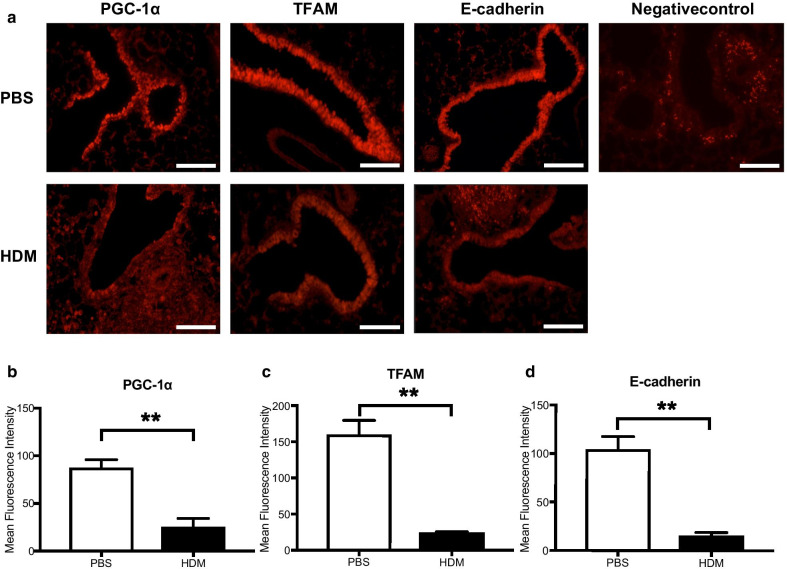
Expression of mitochondrial biogenesis markers in the lungs of HDM-treated mice.** a** C57BL/6 mice were intranasally exposed to PBS or HDM extract every other day for 2 weeks and the mice were sacrificed. Lung tissues were removed after fixation and immune-fluorescence staining was performed to investigate the expressions of mitochondrial biogenesis markers (PGC-1α and TFAM) and E-Cadherin in the lungs of the mice. The negative control indicates secondary antibody alone. Representative photographs are shown. Bars: 100 μm. **b**–**d** The mean fluorescence intensity of PGC-1α, TFAM, and E-cadherin were quantified by using ImageJ. Data are expressed as means ± SD (n = 4). **p < 0.01

The expressions of mitochondrial biogenesis markers and junctional proteins were also analyzed by immunofluorescence in the lungs of mice treated with HDM. The immunofluorescence intensities of PGC-1α, TFAM, and E-Cadherin in the epithelial region were significantly reduced in the lungs from HDM-administered mice compared to the lungs from vehicle-treated mice (PGC-1α, *p* < 0.01; TFAM, *p* < 0.01; E-cadherin, *p* < 0.01) (Fig. 2a–d).

### Effect of SRT1720, a PGC-1α activator, on the HDM-induced reduction of junctional protein expression and epithelial barrier dysfunction in BEAS-2B cells and pHBEC

**Fig. 3:**
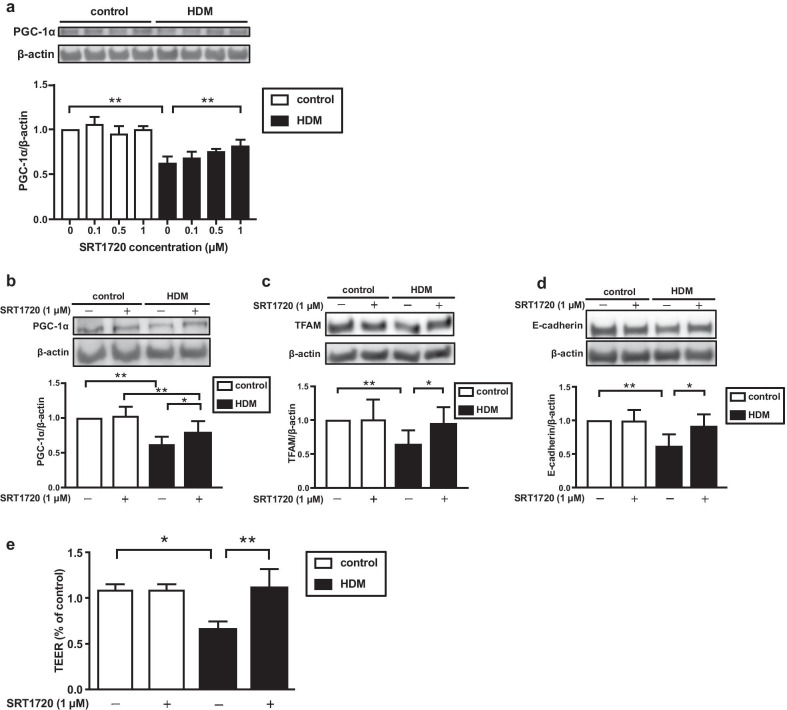
Effect of SRT1720 on mitochondrial biogenesis and airway barrier function mediated by HDM.** a** BEAS-2B cells were treated with various concentrations of SRT1720 or vehicle for 6 h. Subsequently, the cells were stimulated with HDM (100 μg/ml) for 24 h and the cells were harvested. The expression of PGC-1α in the cells was analyzed by western blotting. (n = 3). **p < 0.01. **b**–**d** BEAS-2B cells were treated with vehicle or SRT1720 (1 μM) for 6 h. Subsequently, the cells were stimulated with HDM (100 μg/ml) for 24 h and the cells were harvested. The expressions of PGC-1α, TFAM, and E-cadherin in the cells were analyzed by western blotting (n = 8). **e** Transepithelial electrical resistance (TEER) in primary human bronchial epithelial cells (pHBEC) were measured (n = 4) after the cells were stimulated by HDM (100 μg/ml) or PBS for 24 h with or without SRT1720 (1 μM) using a Millicell ERS-2 voltohmmeter. All data are expressed as means ± SD. *p < 0.05, **p < 0.01

To identify whether a PGC-1α-dependent pathway regulates the expression of junctional proteins in bronchial epithelial cells, we investigated the effects of SRT1720, which is an enhancer of mitochondrial biogenesis by activating PGC-1α, on the HDM-induced reduction in the expression of junctional proteins. SRT1720 (1 μM) significantly restored the HDM-induced reduction in the expression of PGC-1α (*p* < 0.05). Treatment with SRT1720 also significantly restored the HDM-induced reduction in the expression of TFAM (p < 0.05) and E-cadherin (*p* < 0.05) in BEAS-2B cells (Fig. 3a–d). Next, we investigated the effect of SRT1720 on the barrier function by measuring TEER in pHBEC treated with HDM. We used pHBEC instead of BEAS-2B for the detection of TEER because BEAS-2B reportedly showed poor TEER and pHBEC performed well in ALI models and the development of TEER was much better in pHBEC [26]. Treatment with SRT1720 significantly ameliorated the HDM-induced reduction in TEER (*p* < 0.05) (Fig. 3e).

### Effect of SRT1720 on the HDM-induced reduction of junctional protein expression and mitochondrial mass, in BEAS-2B cells by immunocytochemistry

**Fig. 4:**
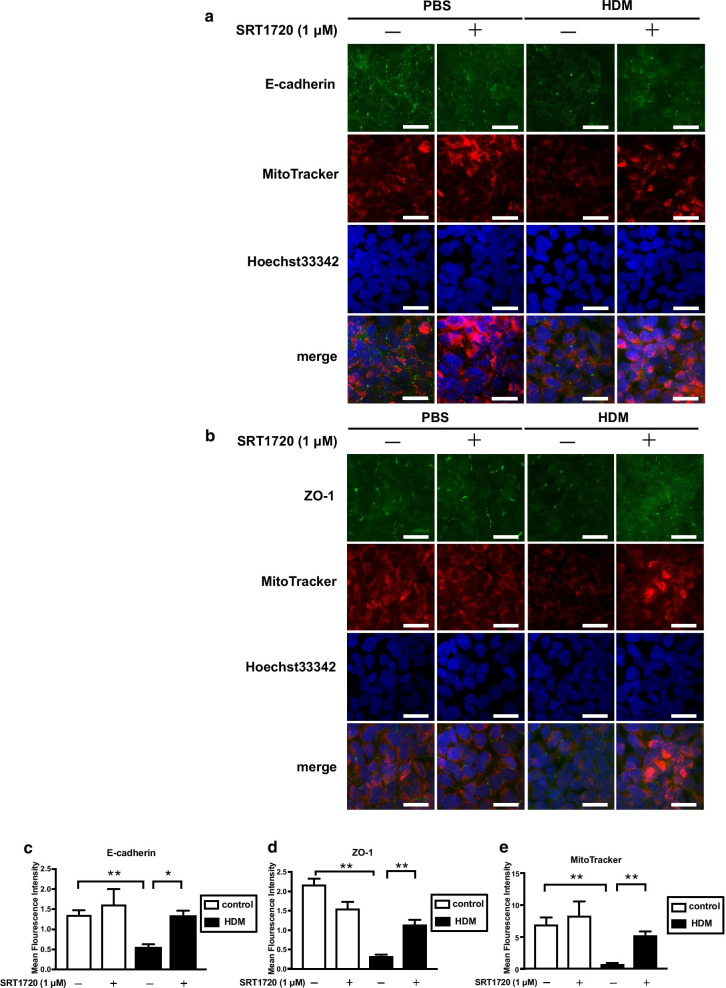
Effect of SRT1720 on mitochondrial mass and expressions of junctional proteins mediated by HDM.** a**,** b** Localization of E-cadherin, ZO-1 and the mitochondrial mass in the cells after exposure to HDM were analyzed by immunofluorescence. Representative confocal microscopic images are shown. The cells were stained with E-cadherin (green), ZO-1 (green), MitoTracker (red), and nuclear counterstain Hoechst33342 (blue). Bars: 100 μm. **c**–**e** The mean fluorescence intensity of E-cadherin, ZO-1, MitoTracker were quantified using ImageJ. Data are expressed as means ± SD. *p < 0.05, **p < 0.01

We also investigated the effect of SRT1720 on the HDM-induced reduction in the expression of junctional proteins in BEAS-2B cells by immunocytochemistry. Treatment with SRT1720 also restored the HDM-induced reduction in the intensity of junctional proteins expression as confirmed (Fig. 4a–d). Next, to investigate how HDM and SRT1720 affect mitochondrial mass, the mitochondrial mass in BEAS-2B cells was analyzed by immunofluorescence using a MitoTracker Red probe. HDM reduced the mitochondrial mass in the cells, which was restored by treatment with SRT1720 (Fig. 4a, b and e).

### Effect of SRT1720 on the HDM-induced release of IL-33 in BEAS-2B cells

Type 2 immune responses to allergen exposure play crucial roles in the pathogenesis of allergic asthma, which is initiated by epithelium-derived cytokines including IL-33, IL-25 and thymic stromal lymphopoietin (TSLP) [27–29]. We investigated how HDM and SRT1720 affect IL-33 release from BEAS-2B cells. HDM significantly enhanced the release of IL-33 in the supernatant of BEAS-2B cells and the concentration of IL-33 was the highest at 30 min after the administration of HDM (Additional file 1: Figure S3a). SRT1720 had no preventive effect on the release of IL-33 initiated by HDM (Additional file 1: Figure S3b).

### The role of PAR2 and TLR4 signaling in the HDM-induced reduction of PGC-1α and junctional protein expression in BEAS-2B cells

**Fig. 5:**
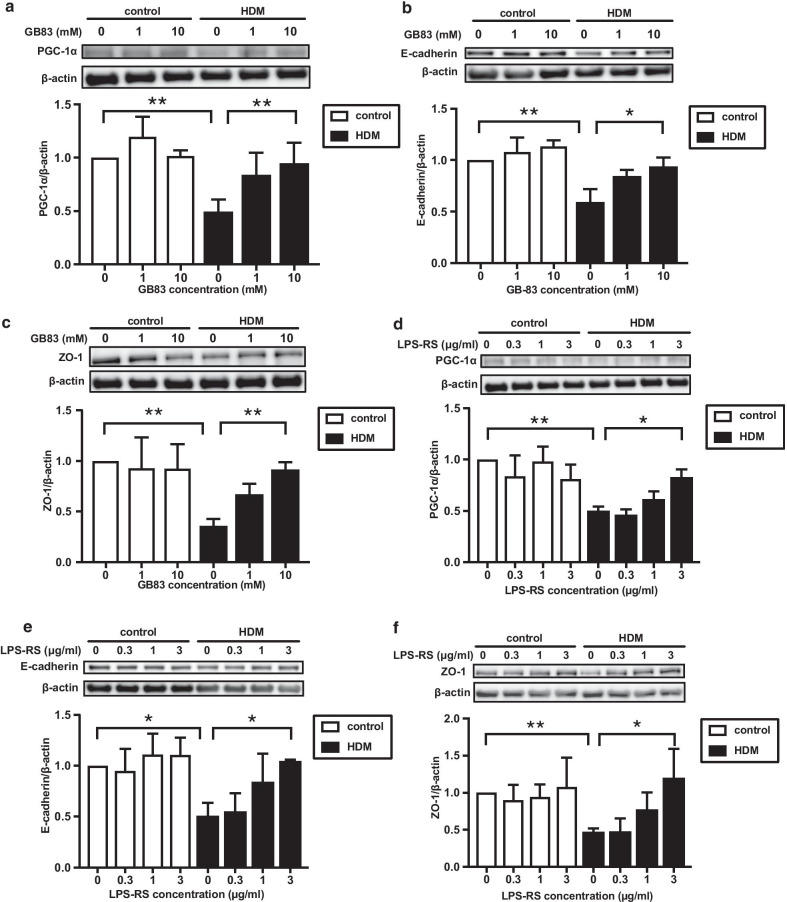
Effects of PAR2 and TLR4 inhibitors on HDM-mediated downregulation of mitochondrial biogenesis and junctional proteins.** a**–**f** BEAS-2B cells were treated with various concentrations of GB83, a PAR2 inhibitor or LPS-RS, a TLR4 inhibitor for 30 min. Subsequently, the cells were stimulated with HDM (100 μg/ml) for 24 h and the cells were harvested. The expressions of PGC-1α, E-cadherin, and ZO-1 in cells were analyzed by western blotting. (LPS-RS, n = 3; others, n = 4). Data are expressed as means ± SD. *p < 0.05, **p < 0.01

HDM contains both proteases and endotoxin, which activate PAR2 and TLR4, respectively. To determine which pathway regulates the HDM-induced reduction in PGC-1α and the subsequent disruption of junctional proteins, we investigated the effects of blockade of PAR2 and TLR4 on the HDM-induced decrease in the expression of PGC-1α and junctional proteins using GB83, a PAR2 inhibitor, and LPS-RS, a TLR4 inhibitor. GB-83 significantly blocked the HDM-induced reduction in the expression of PGC-1α (*p* < 0.01), E-cadherin (*p* < 0.05) and ZO-1 (*p* < 0.01) (Fig. 5a–c) compared to vehicle treatment. Interestingly, LPS-RS also blocked the HDM-induced reduction in the expression of PGC-1α (*p* < 0.05), E-cadherin (*p* < 0.05) and ZO-1 (*p* < 0.05) (Fig. 5d–f).

### Effect of protease inhibitors on the HDM-induced decrease of PGC-1α and E-cadherin expressions in BEAS-2B cells

**Fig. 6:**
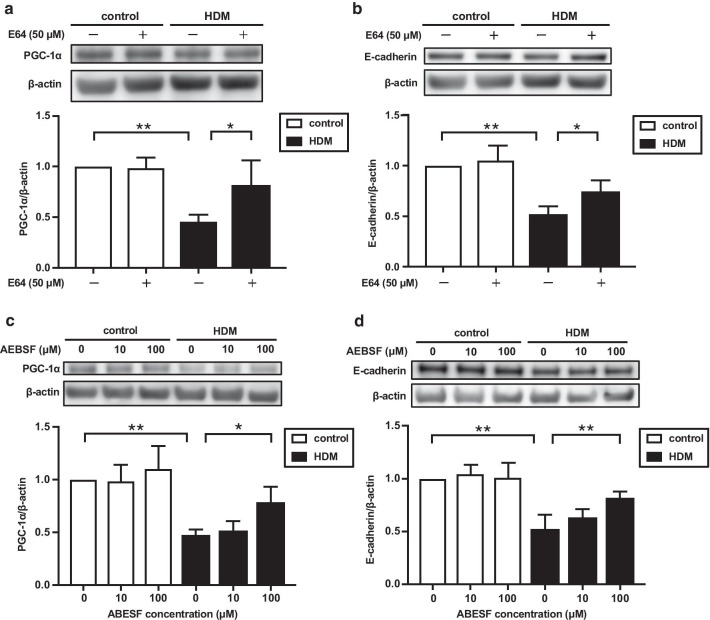
Effects of protease inhibitors on HDM-mediated downregulation of mitochondrial biogenesis and junctional proteins. **a**–**d** BEAS-2B cells were treated with E64 (50 μM) or AEBSF (10 and 100 μM) for 30 min. Subsequently, the cells were stimulated with HDM (100 μg/ml) for 24 h and the cells were harvested. The expressions of PGC-1α and E-cadherin were analyzed by western blotting. (n = 4). Data are expressed as means ± SD. *p < 0.05, **p < 0.01

Serine protease and cysteine protease are present in HDM excrement and cleave junctional proteins directly or indirectly via PAR2 activation. To determine the role of protease activity in the HDM-mediated barrier disruption, we investigated the effects of protease inhibitors on the expression of PGC-1α and E-cadherin in BEAS-2B cells treated with HDM. E64, a cysteine protease inhibitor, significantly blocked the HDM-induced reduction in the expression of PGC-1α (*p* < 0.05) (Fig. 6a), and E-cadherin (*p* < 0.05) (Fig. 6b). AEBSF, a serine protease inhibitor, also significantly prevented the HDM-induced reduction in the expression of PGC-1α (*p* < 0.05) (Fig. 6c) and E-cadherin (*p* < 0.01) (Fig. 6d).

### Effect of corticosteroids on the expression of mitochondrial biogenesis markers and junctional proteins in BEAS-2B cells

**Fig. 7:**
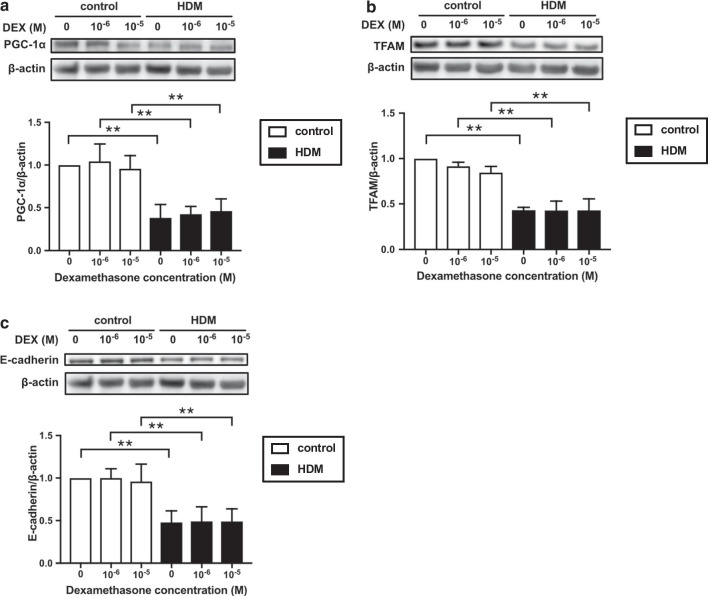
Influence of corticosteroids on HDM-mediated downregulation of mitochondrial biogenesis and junctional proteins.** a–c** BEAS-2B cells were treated with various concentrations of dexamethasone for 24 h. Subsequently, they were treated with HDM (100 μg/ml) and the cells were harvested. Expressions of PGC-1α, TFAM and E-cadherin were analyzed by western blotting (n = 3). Data are expressed as means ± SD. *p < 0.05, **p < 0.01

As inhaled corticosteroids are the central medication for the management of patients with asthma, there is concern about the influence of corticosteroids on the expression of mitochondrial biogenesis markers and the composition of junctional proteins in the airways of asthmatic patients. To address this issue, we investigated whether corticosteroids can affect the expression of markers of mitochondrial biogenesis and junction protein in BEAS-2B cells. Dexamethasone had no significant effect on the baseline expressions of PGC-1α, TFAM and E-cadherin in the cells (Additional file 1: Figure S4a–c). In addition, dexamethasone had no preventive effect on the HDM-induced reduction in the expression of PGC-1α, TFAM and E-cadherin (Fig. 7a–c).

## Discussion

In the current study, HDM, a common aeroallergen to which asthmatic patients are sensitized, decreased the expression levels of the regulators concurrently with the reduction in the amounts of proteins related to the formation of epithelial junctional complex. Activation of PGC-1α by SRT1720 restored the HDM-induced mitochondrial loss and reduction in the expression of E-cadherin, leading to recovery of the airway barrier function. Previous studies demonstrated that the activation of SIRT1/PGC-1α dependent pathway by SRT170 upregulated tight junction molecules in porcine intestinal epithelial cells [30] and in a murine colitis model [31]. These data support our findings, highlighting the important role of SIRT1/PGC-1α pathway in maintaining the epithelial barrier function. The current study suggests a novel mechanism of aeroallergen-mediated airway barrier dysfunction.

In the context of the pathophysiology of asthma, a previous report showed that the up-regulation of PGC-1α in bronchial smooth muscle from asthmatic patients [32] and that non-immune immunoglobulin E (IgE) increased the PGC-1α mRNA and protein levels in primary human airway smooth muscle cells through the activation of phosphatidylinositol 3-kinase (PI3K)-Akt pathway [33], suggesting their involvement in airway wall remodeling. These findings suggest that activation of PGC-1α may cause exacerbation of the asthma pathology. Alternatively, the airway epithelial barrier dysfunction propagates the infiltration of inflammation into the submucosa and impairs the process of epithelial repair. Therefore, the airway epithelial barrier dysfunction is profoundly involved in the progress of remodeling [34]. Thus, SRT1720 may contribute to the prevention of remodeling due to the barrier repair effect on airway epithelium. The role of PGC-1α in the pathogenesis of asthma might depend on cell types in the lung. The effect of SRT1720 on bronchial fibroblasts and airway smooth muscle cells treated with HDM should be investigated to clarify how activation of PGC-1α or SRT1720 affect the airway remodeling.

The innate immune system plays critical roles in the pathogenesis of asthma by recognizing allergens through pattern-recognition receptors (PRRs) including TLRs and PARs, and by providing an early warning system through the production of cytokines and danger signals in bronchial epithelial cells [35, 36]. Many human allergens including HDM, fungi, pollen, and cat contain protease activity and can disrupt tight junction proteins through direct proteolytic cleavage [37]. Furthermore, proteases in HDM activate PAR2 resulting in the loss of E-cadherin in human bronchial epithelial cells [38]. HDM allergens also contain endotoxin and activate TLR4 in bronchial epithelial cells to induce asthmatic features including the release of epithelium-derived cytokines such as IL-25 and IL-33 [39]. Therefore, we investigated the effects of antagonists against PAR2 and TLR4 on the HDM-induced reduction in PGC-1α and tight junction proteins to determine which signaling is responsible. Interestingly, both antagonists significantly prevented the repression of PGC-1α and E-cadherin in HDM-treated BEAS-2B cells. A previous report demonstrated that LPS triggered the activation of NF-κB and the subsequent production of pro-inflammatory cytokines through the cooperative activation of PAR2 and TLR4 in HEK293T cells [40], which is consistent with our findings. It has been reported that the inhibition of TLR4 reverses the decreased expression of PGC-1α in the renal tubules of diabetic mice, suggesting that the TLR4-dependent pathway could be the upstream pathway of PGC-1α [41]. Meanwhile, protease inhibitors were shown to block the HDM-induced reduction in the expression of PGC-1α in this study. Taken together, both TLR4- and PAR2-mediated signaling pathways might be involved in the HDM-induced airway barrier disruption via the regulation of PGC-1α. Details of the interactions between TLR4 and PAR2 during mitochondrial biogenesis and maintenance of the airway barrier function remain unclear and further study is needed to clarify such interactions.

HDM Der p 1 is suggested to directly cleave extracellular domain of tight junctions which initiates intracellular processing of junctional constituents [42]. In addition, Der p 1 can cause indirect intracellular cleavage of tight junctions through a receptor and transduction pathway [42]. As another indirect effect of HDM, ﻿it was demonstrated that Der p 1 exposure decreased the expressions of epithelium tight junction proteins such as ﻿claudin-1 and junction adhesion molecule-A (JAM-A) ﻿in sinonasal cells [43], which is consistent with our results. The current study expands the previous data and showed the contribution of PAR-2 and TLR4 in the HDM-mediated reduction in tight junction proteins. Furthermore, HDM reportedly induced the redistribution of E-cadherin via epidermal growth factor receptor (EGFR)-dependent activation of PAR-2 in human bronchial epithelial cells [44]. Der p 1 has been also shown to suppress the expression of connexin 26, which is involved in the induction and maintenance of tight junction proteins, through PAR-2 activation [45]. All these processes lead to the increased permeability of epithelial cells. Thus, cooperation of direct and indirect routes play a key role in the HDM-mediated barrier dysfunction (Additional file 1: Figure S5).

HDM increased the release of IL-33 in the supernatant, but SRT1720 had no significant effect on the IL-33 release in the current study. Heyen and coworkers demonstrated that IL-33 signaling downregulated E-cadherin expression on pulmonary epithelial cells [46]. IL-33 binds to its primary receptor ST2, leading to the activation of NF-κB or Th2 immune responses. Our results suggest that the initial release of IL-33 caused by HDM may be independent of the PGC-1α pathway. However, SRT1720 ameliorated the HDM-mediated downregulation of the epithelial barrier, and SRT1720 may inhibit the downstream signaling of IL-33/ST-2 interaction. This might be a novel target of Th2 responses. To clarify this, further study is needed to investigate the effect of SRT1720 on Th2 responses in an animal model.

PGC-1α is activated by deacetylation by SIRT1 and phosphorylation by AMPK [47]. Thus, SIRT1 activation enhances PGC-1α-dependent transcriptions. The effect of HDM on SIRT1 expression in BEAS-2B cells remains unknown. The current study demonstrated that HDM downregulated PGC-1α expression thorough TLR4. Furthermore, LPS, a ligand for TLR4, reportedly decreased the expression of SIRT1 in k562 cells [48]. Irisin reportedly improved the LPS-induced alveolar epithelial barrier dysfunction by activating AMPK/SIRT1 pathways in A549 cells and lung tissue of the acute lung injury mouse model [49]. These data suggest that HDM may decrease PGC-1α expression through a TLR4-SIRT1 dependent pathway. The role of AMPK and proteases in addition to SIRT1 expression during the HDM-mediated PGC-1α downregulation together with the detailed mechanisms should be revealed in a future study.

Oxidative stress and nitrative stress play an important role in pathogenesis of asthma. The activation of TLRs including TLR4 leads to overproduction of ROS and reactive nitrogen species (RNS), which can cause the production of proinflammatory mediators and tissue damage [50–52]. Excessively produced ROS have been known to inhibit SIRT1 expression [53, 54]. Exposure to ROS triggered intestinal injury, mitophagy activation and suppressed SIRT1/PGC-1α pathway [30]. Superoxide, one of the ROS, reacts with nitric oxide produced by iNOS during inflammation, leading to the formation of RNS including peroxynitrite [55]. iNOS is shown to cause SIRT1 S-nitrosylation [56]. It has been reported that ADMA increased iNOS activity and production of peroxynitrite in epithelial cells [57]. IL-4 reportedly increased ADMA accumulation in BEAS-2B cells, and in IL-4 plus ADMA-treated cells, expressions of PGC-1α and TFAM were decreased [12]. Our study demonstrated that HDM increased iNOS expression in BEAS-2B cells. These findings suggest that oxidative and nitrative stress can contribute to HDM-mediated PGC-1α downregulation through TLR4 activation.

The present study demonstrated that the mitochondrial mass was decreased by HDM, which was prevented by SRT1720. The mitochondrial mass is determined by the balance of biogenesis and degradation [58, 59]. The activation of mitophagy also reportedly leads to loss of the mitochondrial mass [60, 61]. The present study demonstrated that HDM decreased PGC-1α expression and SRT1720 reversed the HDM-induced loss of the mitochondrial mass in BEAS-2B cells. Meanwhile, HDM increased the expression of PINK1, a marker of the mitophagy. These data suggest that HDM can reduce the mitochondrial mass through the downregulation of mitochondrial biogenesis or the imbalance between mitochondrial biogenesis and mitophagy. PGC-1α-dependent pathway may be the responsible mechanism for loss of mitochondrial mass in this study. However, other markers of mitophagy such as parkin and BCL2-interacting protein 3 (BNIP3), mitochondrial metabolism, and the balance between mitochondrial fusion and fission were not investigated in this study. Further study is needed to clarify the precise mechanisms by which HDM decreases mitochondrial mass and impairs mitochondrial function.

In the current study, we showed that corticosteroids administration did not affect the expression of mitochondrial biogenesis markers, E-cadherin expression, and their HDM-induced reduction in BEAS-2B cells. From these results, it may be considered that the effects of corticosteroids on mitochondrial biogenesis in asthmatic airway epithelial cells are negligible.

A limitation of the current study is that we used only one HDM species, *Dermatophagoides pteronyssinus*, for evaluating the effect of aeroallergen on mitochondrial biogenesis markers and airway epithelial function. Der p 1 and Der p 2 from *Dermatophagoides pteronyssinus* are the dominant allergens in asthmatic patients and have been widely used in experimental airway allergy models. Der p 1 is a cysteine protease allergen and is known to disrupt intracellular tight junction and promote the release of cytokines and alarmins such as TSLP, IL-33 and IL-25 by the protease activity during initial sensitization [62, 63]. Der p 2 is known to mimic MD-2, which facilitates signaling through TLR4, leading to airway inflammation [64]. The current study expands previous findings and demonstrated a novel effect of *Dermatophagoides pteronyssinus* on airway epithelial cells. In addition to *Dermatophagoides ptronyssinu*, *Dermatophagoides farinae* and *Blomia tropicalis* also represent important HDM species [65] and contain different kinds of allergens. Some of them cross react with allergens from *Dermatophagoides pteronyssinus* and many of them are proteases. Therefore, other HDM species may have similar effects on epithelial cells as the allergens from HDM used in this study. However, IgE-binding responses are different depending on each allergen [66]. There are some HDM allergens that have unique effects on airway epithelial cells. Der f 1, an allergen of *Dermatophagoides farinae*, is reported to induce pyroptosis and IL-1β secretion via NLRP3-capase-1 inflammasome in HBEC [67]. Furthermore, there might be a significant difference in the response to HDM between animal models and in vitro culture models. In in vitro models, the effect of molecules on only one isolated cell type can be investigated. whereas the interplay between many different cell types is crucial for cellular homeostasis in vivo [68]. Especially, immunological responses where immune cells such as lymphocytes, innate lymphoid cells and dendritic cells participate can influence HDM-mediated signaling. Although we confirmed that HDM administration decreased expressions of PGC-1α, TFAM and E-cadherin both in vitro and in vivo, the effect of SRT1720 in vivo HDM model needs to be examined. It should also be clarified how immune cells contribute to the HDM-mediated PGC-1α downregulation and how SRT1720 affects immune cells in the future study.

## Conclusion

In conclusion, the current study demonstrated the interaction between innate immunity and mitochondrial biogenesis in the context of inhaled allergen-induced airway barrier dysfunction, an initial feature in the development of asthma. The PAR2/TLR4/PGC-1α-dependent pathway might be a novel and promising therapeutic target for bronchial asthma as, so far, no effective medication for restoring the airway barrier function has been established. Regulating this pathway could inhibit allergic reaction in airways driven by aeroallergens because restoration of the airway barrier function can prevent the penetration of external stimuli, which would suppress the cytokine release that activates type 2 immune reactions. Although further study is needed to confirm the beneficial effect of PGC-1α activator on immunological reactions and to determine the molecules involved in the pathway, this approach might be applicable to a broad array of allergic diseases including allergic rhinitis and atopic dermatitis in addition to bronchial asthma.

## Supplementary Information

**Additional file 1. **Additional figures.

## Data Availability

The datasets used and analysed during the current study are available from the corresponding author on reasonable request.

## Abbreviations

AEBSF: 4- (2-Aminoethyl) benzenesulfonyl fluoride hydrochloride
HDM: House dust mite
LPS-RS: Lipopolysaccharide from Rhodobacter sphaeroides
NRF1: Nuclear respiratory factor 1
PAR: Protease activated receptor
PGC-1α: Peroxisome proliferator-activated receptor γ coactivator-1α
PRRs: Pattern-recognition receptors
Sirtuin 1: SIRT1
TEER: Transepithelial electrical resistance
TFAM: Mitochondria transcription factor A
TLR: Toll-like receptor
ZO-1: Zonula occludens-1
