# Correction to: PGC-1α regulates airway epithelial barrier dysfunction induced by house dust mite

**DOI:** 10.1186/s12931-021-01672-5

**Published:** 2021-03-08

**Authors:** Tsutomu Saito, Tomohiro Ichikawa, Tadahisa Numakura, Mitsuhiro Yamada, Akira Koarai, Naoya Fujino, Koji Murakami, Shun Yamanaka, Yusaku Sasaki, Yorihiko Kyogoku, Koji Itakura, Hirohito Sano, Katsuya Takita, Rie Tanaka, Tsutomu Tamada, Masakazu Ichinose, Hisatoshi Sugiura

**Affiliations:** 1 Department of Respiratory Medicine, Tohoku University Graduate School of Medicine, 1-1 Seiryo-machi, Aoba-ku, Sendai, 980-8574 Japan; 2 Department of Respiratory Medicine, Sendai City Hospital, Sendai, Japan; 3 Department of Respiratory Medicine, Osaki Citizen Hospital, Osaki, Miyagi Japan

## Correction to: Respir Res (2021) 22:63 https://doi.org/10.1186/s12931-021–01663-6

Following publication of the original article [1], we were notified that the supplementary file was incorrect.

The original article has been corrected.
