# Supplementary material for: PGC-1α regulates airway epithelial barrier dysfunction induced by house dust mite
Source: Respir Res. 2021 Feb 19;22:63. doi: 10.1186/s12931-021-01663-6 (PMC7893966; doi:10.1186/s12931-021-01663-6)
Supplement: Supplementary file 1 — Additional file 1. Additional figures. [file 12931_2021_1663_MOESM1_ESM.docx]

**Supplementary figure legends**

**Figure S1. Effect of HDM on expression of mitochondrial transcriptional factor A (TFAM) in BEAS-2B cells.**

BEAS-2B cells were treated with various concentration of HDM for 24 h (A) or with HDM (100 μg/ml) for up to 48 h (B). Subsequently, the cells were harvested and the expression of TFAM in the cells was analyzed by western blotting. Data are expressed as means ± SD (n = 3). *p < 0.05, **p < 0.01.

**Figure S2. Effect of HDM on expressions of PINK1 and inducible nitric oxide synthase (iNOS) in BEAS-2B cells.**

BEAS-2B cells were treated with 100 μg/ml of HDM or PBS for 24 h. Subsequently, the cells were harvested and the expression of PINK1 (A) and iNOS (B) in the cells was analyzed by western blotting. Data are expressed as means ± SD (n = 4). *p < 0.05, **p < 0.01.

**Figure S3. Effect of SRT1720 on HDM-induced interleukin (IL)-33 release in the supernatant of BEAS-2B cells.**

BEAS-2B cells were treated with HDM (100 μg/ml) for up to 240 min. Subsequently, the supernatant was harvested, and the concentration of IL-33 in the supernatant was determined using Enzyme Linked Immunosolvent Assay (ELISA) kit (A). BEAS-2B cells were treated with SRT1720 (1 μM) or PBS for 6 hours. Subsequently, the cells were stimulated with HDM (100 μg/ml) for 30 min and the supernatant was harvested. The concentration of IL-33 in the supernatant was determined using an ELISA kit (B). Data are expressed as means ± SD (n = 3). **p < 0.01. N.S. = not significant.

**Figure S4. Influence of corticosteroids on HDM-mediated downregulation of mitochondrial biogenesis and junctional proteins.**

BEAS-2B cells were treated with various concentrations of dexamethasone for 24 h and then harvested. Expressions of PGC-1α (A), TFAM (B) and E-cadherin (C) were analyzed by western blotting. Data are expressed as means ± SD (n = 3).

**Figure S5. Suggested role of direct and indirect effects of HDM in epithelial barrier disruption.**

HDM directly cleaves the tight junction proteins by the proteases. HDM indirectly affects the expression and distribution of tight junction proteins through a receptor-dependent pathway including PAR-2 and TLR4. The current study suggests that activation of PAR-2 and TLR4 by HDM can inhibit the activation of PGC-1α and, consequently, cause decrease in mitochondrial biogenesis and the expression of tight junction proteins. Both direct and indirect routes are incorporated into the process of epithelial barrier disruption, leading to increased epithelial permeability.

HDM: house dust mite; PGC-1α: peroxisome proliferator-activated receptor gamma coactivator 1 alpha; NRF1: nuclear respiratory factor1; TFAM: mitochondria transcription factor A; TLR4: Toll-like receptor 4; PAR2 protease activated receptor 2; ac: acetylated.
